# Four successful pregnancies in a patient with Fontan palliation and congenital heart disease: a case report

**DOI:** 10.1186/s13019-017-0678-1

**Published:** 2017-11-28

**Authors:** Khalid S. Al Najashi, Syed Mehdi, Shazia Mohsin, Merna Atiyah, Hafez A. Abdullah, Jassim Abdulhameed, Ahmed M. Al Zahrani

**Affiliations:** Prince Sultan Cardiac Center, PO Box 7897, 11159 Riyadh, Kingdom of Saudi Arabia

**Keywords:** Fontan circulation, Single ventricle, Pregnancy, Congenital heart disease

## Abstract

**Background:**

Fontan is a palliative procedure in patients with single ventricle. Single ventricle supports systemic cardiac output and pulmonary blood flow is passively directed to the right pulmonary artery. Women with Fontan palliation are reported to have increased maternal risk during pregnancy. There are few reports of successful pregnancies in such cases. However data on these pregnancies is lacking, we consider this to be the first reported from kingdom of Saudi Arabia.

**Case presentation:**

We present a 35-year-old woman from the Kingdom of Saudi Arabia who had Fontan surgery and who had four successful pregnancies and multiple miscarriages. She delivered live, low birth weight neonates.

**Conclusion:**

This report provides an anecdotal evidence that pregnancy can be tolerated in an adequate Fontan patient with successful outcomes.

## Background

Fontan as a palliation for single ventricle (SV) was first described in 1971 by Fontan and Baudet [1]. They highlighted the role of SV palliation in tricuspid atresia to support systemic cardiac output (CO), while allowing pulmonary blood flow (PBF) to flow passively from the right atrium to the pulmonary artery without an interposed ventricle***.***

Women who reach adolescence are able to have successful births after Fontan palliation, despite being counseled against pregnancy because of the high maternal and fetal risks. In pregnancy, normal physiological changes, such as increased blood volume, decreased hematocrit, decreased systemic vascular resistance, increased heart rate, and increased CO can deteriorate the already compromised CO and PBF in Fontan palliation. This leads to arrhythmias, worsening cyanosis, heart failure, and impaired exercise capacity [2]. Fetal complications in the form of intrauterine growth retardation, low birth weight, and preterm birth have also been reported [3].

However, detailed data on these pregnancies are still lacking. We report a case of a 35-year-old woman with Fontan palliation who had four successful pregnancies. This is the first such case to be reported from the Kingdom of Saudi Arabia and provides anecdotal evidence that pregnancy may not necessarily be contraindicated in a Fontan patient.

## Case presentation

We report a 35-year-old Saudi woman with the diagnosis of transposition of the great arteries, ventricular septal defect, severe pulmonary stenosis, a sub-aortic chamber, and a rudimentary right ventricle. She had been diagnosed with these conditions at three years of age and was under regular follow-up at our center. Total cavo-pulmonary anastomosis with the lateral tunnel procedure was performed at the age of 13.5 years. She was kept on Aspirin as antithrombotic treatment for one year after Fontan.

Our patient experienced menarche at 13 years, but had irregular cycles with menorrhagia. Over the subsequent few years she did not report to the hospital. She conceived repeatedly, had five miscarriages, and delivered three low birth weight neonates. All of her previous pregnancies were spontaneous vaginal delivery (SVD) at a peripheral hospital (Table 1). At the time when she presented to us she was pregnant and had three live babies. The first two neonates were full term births the third baby was pre-term baby at 30 weeks of gestation, who was admitted to Neonatal Unit for three weeks for feeding purposes.

**Table 1:** Details of the patient’s pregnancies

Age of patient (years)	Mode of Delivery	Gestational age at delivery (weeks)	Baby sex	Complication after delivery
20	Spontaneous Vaginal Delivery	38	F	NONE
21	Miscarriage	16	_	NONE
22	Miscarriage	23	–	NONE
23	Miscarriage	20	–	NONE
24	Miscarriage	12	–	NONE
27	Miscarriage	9	–	NONE
30	Spontaneous Vaginal Delivery	37 weeks	F	NONE
33	Spontaneous Vaginal Delivery	30 weeks	M	Admitted in neonatal unit for 3 weeks for feeding purposes
35	Cesarean-section	36 weeks	M	NONE

She was on regular antenatal follow-ups at our center in her last pregnancy. Holter was repeated which showed normal sinus. Transthoracic echocardiography was done showing no Fontan pathway obstruction and no fenestration was seen. There was a mild right and left atrio-ventricular valve insufficiency with good SV function. On routine laboratory investigation, she had iron deficiency anemia. Her Hemoglobin was 10.6 g/dl. She was prescribed iron and multivitamin tablets. She received Aspirin throughout her pregnancy.

A fetal echo was performed at 16 weeks’ gestation and showed normal cardiac anatomy for the fetus. Her Electro-cardiogram (ECG) and clinical examination during these antenatal visits were normal. Because of less chance of complications, instrumental vaginal delivery is the preferred mode in these patients according to the Association of European Pediatric Cardiology. However, at 36 weeks of pregnancy, our patient had elective cesarean section (CS) because of obstetric preference. Her aspirin was stopped 48 h prior to CS. She delivered a full-term, low birth weight male neonate who weighed 2.1 kg. She was reviewed at a later date by the joint cardiology–obstetric team. She was prescribed sub-cutaneous Enoxaparin and Aspirin for six weeks after CS then she was advice to continue on Aspirin 81 mg once a day, iron and multi vitamin tablets. She was counseled in detail regarding contraception and avoiding further pregnancies, and to regularly have follow-up with the adult congenital heart disease team.

At a follow-up visit, 6 weeks after delivery, she was asymptomatic with New York Heart Association functional class II. Her physical examination was unremarkable and oxygen saturation was 93% in room air. N-terminal prohormone of brain natriuretic peptide (248 pg/mL), total protein, and albumin levels were in the normal range.

A chest X-ray showed a normal heart size with normal pulmonary vascular markings. ECG showed normal sinus rhythm with a heart rate of 70 beat per minute and normal QRS duration. A transthoracic echocardiograph showed a patent Fontan pathway with mild left-sided atrio-ventricular valve insufficiency and good SV function. A treadmill exercise test was performed and she tolerated 8 min of exercise, with 85% achievement of her predicted values. Her 24-h Holter recording was negative for arrhythmia and the average heart rate was 80 bpm.

## Discussion

Fontan palliation is performed with the aim of normalizing the volume load of a Functional SV by only ejecting systemic CO. This results in elevated right atrial and systemic venous pressure, leading to protein-losing enteropathy, thromboembolism, and heart failure. The high atrial wall tension results in a high propensity of atrial tachycardia in these patients. With advancements in the field of pediatric cardiology, patients with SV survive well into adulthood, and pregnancy might be considered. Cases of infertility and delayed menarche in patients with Fontan palliation have been reported. However successful pregnancies have also been reported [3, 4]. The age of menarche was described as ranging between 12 to 13.5 years in a review in different countries by Drenthen et al. [3]. Our patient reached menarche at 13 years old, despite having an irregular menstrual cycle, and she had multiple conceptions.

Complications that are reported in SV palliation during pregnancy could be due to a fall in systemic vascular resistance and increase in CO. A 30–50% increase in intravascular volume occurs in normal pregnancy during the second trimester and peaks early in the mid-third trimester. CO increases to accommodate for these volume changes by an elevation in heart rate of approximately 10 to 20 beats per minute and an increase in stroke volume [4]. Fontan patients have limited ability to cope with these physiological demands. Data regarding pregnancy outcomes in patients who have the Fontan procedure are limited, but maternal and fetal complications have been reported. Atrial tachycardia is the most common complication because of right atrial tissue exposure to higher than normal pressure and incisional scar tissue formation [3, 5]. Deterioration in New York Heart Association class (15%) is also commonly reported because of additional circulatory overload, although it is relatively benign and is rapidly resolved after delivery [3]. Additionally, Fontan patients are mildly cyanotic, which might decrease fertility and cause a higher rate of miscarriage. Spontaneous miscarriage has also been reported more frequently in patients with Fontan palliation compared with a miscarriage rate of 15–20% in all pregnancies [6]. Among nine conceptions, five in our patient resulted in miscarriage. This finding is consistent with data by Pundi et al. [7], who reported a 50% rate of miscarriage in their cohort of 70 patients.

Obstetric complications in women with Fontan palliation are also a major concern. Our patient had four successful pregnancies, and one was near term and one was preterm. All four pregnancies resulted in low birth weight neonates.

Similarly, Drenthen et al. [3] and Gouton et al. [4] reported that 28 to 69% of Fontan pregnancies were preterm deliveries. However, Collin et al. [5] reported that, in 282 Fontan pregnancies, 1/5 neonates were preterm.

The Association of European Pediatric Cardiology stated that successful pregnancy outcomes in Fontan patients is possible in selected patients with preconception analysis, individual risk assessment and careful multidisciplinary evaluation, and intensive monitoring during and after pregnancy and at delivery [8]. They recommend assisted vaginal delivery as the preferred mode of delivery in these patients. This mode of delivery reduces the chances of complications with SVD and CS. The Valsalva method in SVD can further decrease already compromised PBF, leading to hypoxemia. General anesthesia and positive pressure ventilation in CS can decrease systemic vascular resistance and CO, thereby aggravating heart failure and hypoxemia Our patient decided to undergo CS because of obstetric indications. She was monitored throughout and immediately after CS. She did not develop any major complications.

Elkayam et al. [9] recommended SVD as the preferred mode for women with SV after Fontan and CS should be considered if there are fetal or maternal indications. Collin et al. [5] reported that 26% of SV pregnancies had delivery via CS with no adverse outcomes, as also reported by Pundi et al. in their cohort [7].

Comprehensive evaluation including Doppler echocardiography to assess ventricular function and Fontan pathway patency is advisable. Our patient had detailed cardiac evaluations in the antenatal period and after delivery, and these evaluations showed normal parameters.

A Fontan patient’s ability to cope with increased physiological demands in pregnancy depends on the ability to increase CO, despite elevated venous pressure. Patients with Fontan palliation who are clinically and hemodynamically stable before pregnancy will most likely better tolerate pregnancy and delivery with complete evaluation and monitoring in a cardiac center with a multidisciplinary team [10]. Zentner et al. [11] reviewed a cohort of 101 pregnant Fontan women and did not find any maternal mortality during pregnancy. Therefore, pregnancy should not be contraindicated in wellfunctioning patients with Fontan palliation. However, the long-term effects of pregnancy on the Fontan heart remains to be determined [11].

## Conclusion

Although there have been previous reports of Fontan patients with multiple pregnancies, this is the first report from Saudi Arabia. This case report provides anecdotal evidence that pregnancy may not necessarily be contraindicated in Fontan with adequate repair without long-term complications. If socio-cultural pressure overrides conventional medical advice against pregnancy, these patients can be successfully managed by individual risk assessment and careful multidisciplinary evaluation during and after pregnancy.

## Abbreviations

CO: Cardiac output
CS: Caesarian section
ECG: Electro-cardiogram
PBF: Pulmonary blood flow
SV: Single ventricle
SVD: Spontaneous vaginal delivery
